# Adherence to the Mediterranean Diet Is Associated with Physical Activity, Self-Concept and Sociodemographic Factors in University Student

**DOI:** 10.3390/nu10080966

**Published:** 2018-07-26

**Authors:** Félix Zurita-Ortega, Silvia San Román-Mata, Ramón Chacón-Cuberos, Manuel Castro-Sánchez, José Joaquín Muros

**Affiliations:** 1 Department of Didactics of Musical, Plastic and Corporal Expression, University of Granada, 18071 Granada, Spain; felixzo@ugr.es (F.Z.-O.); jjmuros@ugr.es (J.J.M.); 2 Department of Nursing, University of Granada, 18071 Granada, Spain; silviasanroman@ugr.es; 3 Department of Integrated Didactics, University of Huelva, 21007 Huelva, Spain; ramon.chacon@ddi.uhu.es; 4 Department of Education, University of Almería, 04120 Almería, Spain

**Keywords:** Mediterranean diet, physical activity, self-concept, socioeconomic factors

## Abstract

(1) Background: The aim of this study was to assess adherence to the Mediterranean diet (MD) and to examine the relationship between MD adherence, physical activity, self-concept, and other sociodemographic factors; (2) Methods: A cross-sectional study (*N* = 597; 18.99 ± 0.64 years) was conducted in a sample of university students from Ceuta, Melilla, and Granada (Spain). Religious beliefs and place of residence were directly reported, while physical activity and adherence to the MD were self-reported using the Physical Activity Questionnaire for Adolescents (PAQ-A) and the Mediterranean Diet Quality Index (KIDMED) respectively. Self-concept was evaluated using the Five-Factor Self-Concept Scale; (3) Results: Of those students reporting high levels of habitual physical activity, 82.3% also reported high adherence to the MD, with 17.7% reporting a medium adherence. Of students reporting no physical activity, 25.7% also reported medium adherence to the MD. No significant associations were found between the MD and religious beliefs. It was observed that the university campus was associated with the level of adherence to the MD (*p* = 0.030), with adherence being lowest in Ceuta and Melilla. Finally, the MD was associated with academic (*p* = 0.001) and physical self-concept (*p* = 0.005); 4) Conclusions: The MD should be promoted to university students, particularly those studying at Ceuta and Melilla, given the present findings of lower MD adherence. In addition, as higher MD adherence was also highlighted with more positive self-concept, its promotion would be beneficial in wider educational contexts.

## 1. Introduction

Considerable research has addressed the beneficial effects of the Mediterranean diet (MD) on life expectancy and quality of life [1], since it has a positive effect on public health, and reduces the risk of suffering cardiovascular diseases, Alzheimer’s, depression, diabetes, and cancer, as well as many others [2,3,4,5,6,7]. In addition, the MD is associated with better cognitive and emotional functioning [6,7]. In addition, higher socioeconomic status and living in the family home is associated with a better quality of diet [8,9,10].

The composition of the MD is based on the consumption of food cultivated around the Mediterranean Sea, which contain a high amount of natural antioxidants. It is characterized by a high intake of olive oil, cereals, fruits, vegetables, and legumes, with also a moderate consumption of fish, eggs, and dairy products. Red and processed meats, saturated fats, and alcoholic drinks are consumed in low amounts [3,11,12]. Following a balanced diet, the MD has been shown to provide essential macronutrients and demonstrates important health benefits, such as improved body composition and the prevention of cardiovascular diseases [4,5,6,10].

The benefits of adhering to a MD have been studied for young people living in Mediterranean areas are shown in countries, such as Italy [13,14], Turkey [15,16], Cyprus and Greece [17,18], Spain [19,20], Lebanon [11], and Croatia [21]. Additionally, studies have been conducted in countries with cultural and linguistic influence, such as Chile [4,10], Brazil [22], or the south of the United States [23].

Arnet describes university students as being in an “emerging adulthood stage [24] during which they possess the biological maturation of an adult, but have not yet achieved comparable psychosocial development [19]. These young adults, often during the first years of study, live outside of the family home, often with negative implications for the quality of their diet [24,25]. Unhealthy habits appearing during this life stage often persist into adulthood, for example, previous research has linked poor nutrition with the use of harmful substances and negative repercussions later in life [25,26].

Diet is one of the cornerstones of a healthy lifestyle, and it is sometimes characterised in university students by a lack of meals and essential foods, and by the intake of large amounts of sugar, alcohol, and saturated fats [27]. These facets may be influenced by the place in which the student lives (e.g., shared flat, halls of residence), with moving away from home to live in the city of the university being a potentially important factor [11,25,28,29]. Further, university students often show reduced levels of physical activity and greater engagement in sedentary behaviour [24]. It should be noted that this is not the case for all students, with one study of students enrolled in nursing and teaching courses showing a higher adherence to the MD [30,31]. Studying subjects related to nutrition may, therefore, be a protective factor for some of the previously identified negative habits.

Self-concept is defined as the collection of beliefs about oneself and is also associated with health behaviours [32]. For instance, diets with an excess of fat and sugar can lead to increased overweight indices and a negative belief about oneself [33,34]. Moreover, several studies have shown that a worse adherence to the MD is associated with lower academic performance and subsequent negative academic self-concept [35], and negative emotions, such as higher levels of stress and anxiety, and a subsequent negative emotional self-concept [36].

Students’ religious beliefs might also have an influence, since the Mediterranean Sea is surrounded by countries that are traditionally Catholic and Islamic [37]. The Islamic religion associated with Arabian culture does not allow the consumption of some food, such as pork or alcohol. It could be thought that Spain, since it is so near to Africa, could have similar food and cooking traditions. However, the norms associated with both regions are very different. The cities of Ceuta and Melilla, for example, are Spanish, but are in Africa, thus, being highly influenced by Arabian culture [38].

Although the MD has been studied in relation to physical activity and healthy lifestyles, research in relation to self-concept, place of residence [24,39], and cultural-religion [38] is scarce. Thus, the present study will present novel data on the MD and its association with sociodemographic, physical, and emotional factors within a sample of university students enrolled in social and health science courses based at three campuses of the University of Granada (Ceuta, Melilla, and Granada).

The present study will address the following research question and associated hypotheses: Is adherence to the MD related to religion, place of residence, physical activity, and self-concept of university students from different parts of Spain?

**Hypothesis 1** **(H1).** *The university students from Ceuta and Melilla will present a lower adherence to the MD. In addition, those who do not live in a shared flat and report Christian beliefs will follow a better-quality diet*.

**Hypothesis 2** **(H2).** *University students reporting higher levels of physical activity will also report greater adherence to the MD*.

**Hypothesis 3** **(H3).** *Higher adherence to the MD will be associated with more positive self-concept both overall and for all its associated dimensions*.

The main aims of the present study are: (a) To assess adherence to the MD within a population of students from Ceuta, Melilla, and Granada (Spain); and (b) to examine the relationship between adherence to the MD, physical activity, self-concept, religion, and place of residence.

## 2. Materials and Methods

### 2.1. Subjects

The present research reports a cross-sectional study with a sample of 636 university students aged between 18 and 20 (18.99 ± 0.64 years) enrolled in education or health-related degree programmes in Granada, Ceuta, and Melilla (Spain). Of these, 39 participants did not meet the inclusion criteria (i.e., incomplete questionnaires, failure to complete informed consent forms) and so could not be included. The final sampled, therefore, consisted of 597 participants (156 males and 441 females): Granada (*n* = 103); Ceuta (*n* = 138); and Melilla (*n* = 356).

### 2.2. Instruments

The Mediterranean Diet Quality Index (KIDMED) [40] was used to determine the level of adherence to the MD. This questionnaire consists of sixteen items that relate to Mediterranean dietary patterns. Four items denote negative connotations with respect to the MD (e.g., do you eat sweets daily?) and are scored as a −1, while twelve items relate to positive connotations (e.g., do you use olive oil for cooking at home?) and are scored as a +1. All items are then summed to produce a total score, which ranges from −4 to 12. Adherence to the MD can then be classified as high (≥8), medium (2–7), or low (≤1). The KIDMED demonstrates reliable psychometric properties using Cronbach’s alpha (α = 0.836).

To estimate physical activity levels, the Physical Activity Questionnaire for Adolescents (PAQ-A) was used, which has been previously validated by Kowalski et al. [41] and adapted to Spanish by Martínez-Gómez et al. [42]. The instrument asks participants to retrospectively report the frequency and type of physical activity engaged in on each of the seven days preceding administration. The questionnaire comprises ten items (e.g., last weekend, how many times did you do sports, dance, or play games in which you were very active?), rated using a five-point Likert scale. Individual items are then summed to create a variable, which describes overall physical activity levels. The PAQ-A demonstrates reliable psychometric properties using Cronbach’s alpha (α = 0.874).

Self-concept was assessed using the Five-Factor Self-Concept Questionnaire (AF-5) [43]. The AF-5 is formed according to the dimensions of academic self-concept (A-SC), social self-concept (S-SC), emotional self-concept (E-SC), family self-concept (F-SC), and physical self-concept (P-SC). The test includes thirty items (e.g., I am a friendly person), which are rated on a five-point Likert scale ranging from never (1) to always (5). Each dimension is described by six items (A-SC: Items 1, 6, 11, 16, 21, 26; S-SC: Items 2, 7, 12, 17, 22, 27; E-SC: Items 3, 8, 13, 18, 23, 28; F-SC: Items 4, 9, 14, 19, 24, 29; P-SC: Items 5, 10, 15, 20, 25, 30). The AF-5 demonstrates reliable psychometric properties using Cronbach`s alpha (α = 0.800). This replicates earlier reports by García and Musitu [43] (α = 0.810).

Socioeconomic factors were directly reported by participants using a self-registration sheet. Participants reported their gender, age, religious belief (Catholic, Muslim, Atheist-Agnostic, and other), and place of residence (family home, shared flat, or university residence).

### 2.3. Procedure

Eligible participants were provided a detailed explanation of the aims and implications of the research. Those who decided to participate then provided written informed consent. Participants were instructed on how to fill out the questionnaires. All tests were conducted during a non-teaching lesson at the student’s university. No incentives were provided for participation. A research assistant attended all data collection sessions to provide guidance on the completion of questionnaires. Ethical principles of the Declaration of Helsinki were adhered to and ethical approval was granted by the Ethics Committee of the University of Granada.

### 2.4. Statistical Analysis

Data were analysed using SPSS^®^ version 24.0 (IBM Corp, Armonk, NY, USA) for Windows. Means for all quantitative variables (self-concept) are reported alongside the standard deviation. Averages and percentages are presented for all qualitative variables (MD, physical activity, gender, religious belief, university campus, and place of residence). Normality of the data was tested using the Kolmogorov-Smirnov test with Lilliefors correction, and homoscedasticity was assessed using the Levene test. The association between variables was determined using chi-square analysis and ANOVA. The level of significance was set at 0.05.

## 3. Results

Table 1 shows the proportion of the sample attending the university campuses of Granada, Ceuta, and Melilla. A total of 636 university students were invited to participate in the study. Of these, 39 students were excluded for not meeting inclusion criteria, leaving a final sample of 597 participants. Of the final sample, 17.3% (*n* = 103) were attending the university campus in Granada, 23.1% (*n* = 138) were attending in Ceuta, and 59.6% (*n* = 356) were attending in Melilla.

As shown in Table 2, adherence to the MD was high, medium, and low for 77.6%, 21.9%, and 0.5% of the sample, respectively. The percentage of participants who reported engaging in physical activity was similar to those reporting not engaging in physical activity (52.8% vs. 47.2%). A total of 54.9% of participants reported being of Catholic faith, 27.5% were atheist or agnostic, and 16.3% identified themselves as Muslim. A total of 63.8% of the participants reported residing with their family, while only 6.7% reported residing in student housing. Self-concept was reported most positively for the dimension relating to family (Mean (M) = 4.36) followed by the social dimension. Academic, emotional, and physical self-concept were rated less positively, with average scores being lower than 3.65 for these dimensions.

As shown in Table 3, there were significant associations between MD adherence and physical activity (*p* = 0.014). Students who reported practicing physical activity were more likely to report a high adherence to the MD (82.3% of the active participants), while only 73.3% of participants who reported not engaging in physical activity reported a high adherence to the MD.

As shown in Table 4, the MD was associated with academic self-concept (*p* = 0.001), which was more positive in students with high MD adherence (M = 3.67) relative to those reporting only a medium adherence (M = 3.45). The same tendency was observed with regards to physical self-concept (*p* = 0.005), with higher scores reported by students with high MD adherence (M = 3.39) compared with those who had a medium adherence (M = 3.16). No differences were found with regards to social, emotional, and family self-concept (*p* ≥ 0.050).

Finally, no significant differences (*p* ≥ 0.050) were found in the associations between MD, religious belief, and place of residence (Table 5). It was observed that the university campus was associated with adherence to the MD (*p* = 0.030). The percentage of students who had a high adherence was 90.2% in Granada, 74.7% in Ceuta, and 72.5% in Melilla. Medium adherence was reported by 10.7% in Granada, 23.9% in Ceuta, and 26.7% in Melilla.

## 4. Discussion

The main objective of the present study was to identify the relationships between adherence to the MD, physical activity, self-concept, and selected sociodemographic factors. High adherence to a MD was reported by almost 80% of the included participants. One factor that could contribute to this high level of adherence is the relatively young age of the participants (none of whom were older than 20 years of age), making it likely these participants were still highly influenced by their family with regards to their eating behavior, a suggestion supported by previous research conducted by San Mauro et al. [44]. On the other hand, previous research conducted with samples of older university students has failed to support these suggestions [26,29,45].

More participants in the present study were attending the campus in Melilla than Ceuta or Granada. At this particular campus, all of the degrees pertained to the fields of social sciences or health. While the campus of Granada is the biggest of the three, it also has the greatest variety of available courses. The present study chose to focus on students studying courses previously identified by Rodrigo et al. [31] as being disciplines with likely associations with adherence and understanding of the MD. Further, it is also important to highlight the greater participation of women in the present study, which is because these areas of study are more popular amongst women than men [29].

Findings relating to the practice of physical activity are similar to those obtained in other studies, which have shown physical activity engagement to decrease upon reaching adulthood and starting university. This may at least be partly caused by the change in social tendencies and the development of habits leading to weight gain, stress, and anxiety [46,47,48]. This life period is, therefore, critical and the promotion of healthy habits in relation to nutrition and physical activity is necessary [49].

Associations were established between adherence to the MD and the practice of physical activity, with the conclusion that those who exercise regularly have better nutritional levels. Students with low adherence to the MD tended also to not engage in physical activity, whilst those typically adhering to a MD diet also show higher levels of physical activity [50,51,52]. One potential explanation for this is that young people who practice physical activity tend to consume a nutritious diet to obtain greater results in terms of performance, body image, or wellbeing [53,54].

In the present study, academic self-concept was higher in students who reported high adherence to the MD than in those who reported low adherence. This suggests that diet influences academic achievement, as has been suggested by Unal et al. [55] who discuss the importance of breakfast and the MD as a contributor to academic success. Since a better academic performance is related to a more positive perception of the individual in the university environment, their academic self-concept could improve [55,56]. In this sense, these results are similar to those reported in other teenage populations [56] and corroborate previous research conducted by Inauen et al. [57], which discussed the benefits of exercise on nutritional behavior.

Contrary to research suggesting an increase in the amount of people who do not practise any religion in Spain [37], Catholicism is still followed in southern areas. However, in the areas of Ceuta and Melilla, the Islamic faith is widely practiced. In recent years, the number of degrees studied at the three university campuses has increased, leading to more students studying locally and living in their family home. Resultantly, the family dimension of self-concept becomes highly valued and has a clear effect on improving nutritional consumption.

In the present study, religious and cultural tendencies were not associated with diet, which is contrary to previous findings reported by Navarro-Prado et al. [38] suggesting that Muslims had a lower quality diet than Christians due to their regular omission of breakfast and dinner. The present study suggests that culinary cultures in the south of Spain and the north of Africa influence one another, thus, masking differences. Age is likely a stronger influence on eating behaviours than place of residence as almost 70% of the participants still lived at home, reducing the variability of this variable. Furthermore, several studies, such as those developed by Donnelly et al. [58] and Bernardo et al. [59], demonstrate that the cost of healthy food inhibits the ability of university students to adhere to a MD.

Higher MD adherence was reported by participants studying at the campus in Granada than the other two included campuses. As this city is inside the Peninsula, it has an enhanced supply of good quality food in comparison to those cities outside of the Peninsula. This, alongside the previously mentioned culture of Arabian food in Ceuta and Melilla, [38], can help to explain the lower adherence to the MD in these regions.

The present study also has a number of limitations. The cross-sectional design prevents any conclusions pertaining to causality from being made. This type of study provides preliminary evidence, which should now be followed by longitudinal research. Other limitations relate to the scales used. For instance, errors caused by poor comprehension of the KIDMED test and the self-report questionnaire were identified, though evidence suggests that such errors should not impede their application [8,60]. Finally, while the PAQ-A demonstrates good internal consistency within samples of university students and Arnet [24] has highlighted similarities during emerging adulthood and this period of adolescence, it should be noted that this instrument has only been validated within samples of adolescents and older adolescents, and so could present adjustment errors within this sample.

In consideration of the limitations and findings of the present study, the following suggestions are made for future research. Future studies should seek to apply the present self-report instruments to more areas of knowledge and within samples of adults. Moreover, the PREDIMED and GPAQ questionnaires may be more appropriately used to measure adherence to the MD and physical activity behaviour in future research as they have been validated amongst young adults. Finally, an intervention using nutritional talks and practical activity should be developed to improve the level of adherence to the MD of university students, particularly in the cities of Ceuta and Melilla. This should also include periodic measurement of psychosocial factors and health indices to ascertain relationships with adherence to the MD.

## 5. Conclusions

Adherence to the MD was related to certain socioeconomic factors, such as the university campus attended, the practice of physical activity, and some dimensions of self-concept. The following conclusions can, therefore, be made regarding the study hypotheses:

- Hypothesis 1 (H1) was partially supported as university students from Ceuta and Melilla reported a lower adherence to the MD. However, statistically significant differences were not found according to the place of residence and religious belief;
- Hypothesis 2 (H2) was completely supported. The results showed the practice of physical activity to be associated with a higher adherence to the MD; and
- Hypothesis 3 (H3) was partially supported, since adherence to the MD was only associated with the physical and academic dimensions of self-concept.

The main conclusions established from the present study were that participating students at the three included campuses typically reported moderate levels of MD adherence, with adherence being higher amongst students at the Granada campus. No relationships were identified between MD and the place of residence or religious belief, while physical activity was positively associated with MD adherence. Finally, adherence to the MD was positively associated with the physical and academic self-concept. Given the relationships observed, the MD should be promoted in university students, with a view to improving psychosocial factors. The lower quality of diet prevalent at the campuses of Ceuta and Melilla highlights these areas as important contexts for intervention. A main strength of the present analysis of the MD in a population with particular social and cultural characteristics is the highlighting of relevant social factors, such as religion, and psychological factors of well-being, such as self-concept. Future research should now build on this to replicate the present study within other populations of relevance, such as children or adolescents, and to include other key factors for health, such as overweight or substance consumption.

## Figures and Tables

**Table 1:** Proportion of the sample (eligible sample, excluded participants, and final sample) attending the university campuses of Granada, Ceuta, and Melilla.

University Campus	Eligible Sample % (*n*)	Excluded Participants % (*n*)	Final Sample % (*n*)
Granada	17.6% (*n* = 112)	23.1% (*n* = 9)	17.3% (*n* = 103)
Ceuta	23.4% (*n* = 149)	28.2% (*n* = 11)	23.1% (*n* = 138)
Melilla	59.0% (*n* = 375)	48.7% (*n* = 19)	59.6% (*n* = 356)
Total	100.0% (*n* = 636)	100.0% (*n* = 39)	100.0% (*n* = 597)

**Table 2:** Characteristics of study sample according to sex.

Variables		Sex		
		All (*n* = 597)	Male (*n* = 156)	Female (*n* = 441)
**MD**	**High**	77.6% (*n* = 463)	33.3% (*n* = 118)	66.7% (*n* = 345)
	**Medium**	21.9% (*n* = 131)	28.2% (*n* = 37)	71.8% (*n* = 94)
	**Low**	0.5% (*n* = 3)	0.6% (*n* = 1)	0.5% (*n* = 2)
**Campus**	**Granada**	17.3% (*n* = 103)	18.4% (*n* = 19)	81.6% (*n* = 84)
	**Ceuta**	23.1% (*n* = 138)	26.8% (*n* = 37)	73.2% (*n* = 101)
	**Melilla**	59.6% (*n* = 356)	28.1% (*n* = 100)	71.9% (*n* = 256)
**Physical Activity**	**Yes**	47.2% (*n* = 282)	39.4% (*n* = 111)	60.6% (*n* = 171)
	**No**	52.8% (*n* = 315)	14.3% (*n* = 45)	85.7% (*n* = 270)
**Religious Belief**	**Catholic**	54.9% (*n* = 328)	25.3% (*n* = 83)	74.7% (*n* = 245)
	**Muslim**	16.3% (*n* = 97)	13.4% (*n* = 13)	86.6% (*n* = 84)
	**Atheist-Agnostic**	27.5% (*n* = 164)	34.1% (*n* = 56)	65.9% (*n* = 108)
	**Other**	1.3% (*n* = 8)	50.0% (*n* = 4)	50.0% (*n* = 4)
**Place of Residence**	**Family Home**	63.8% (*n* = 381)	24.9% (*n* = 95)	75.1% (*n* = 286)
	**Shared Flat**	29.5% (*n* = 176)	26.7% (*n* = 47)	73.3% (*n* = 129)
	**University Residence**	6.7% (*n* = 40)	35.0% (*n* = 14)	65.0% (*n* = 26)
**Self-Concept**	**A-SC**	M = 3.62 SD = 0.601	M = 3.48 SD = 0.600	M = 3.67 SD = 0.595
	**S-SC**	M = 3.91 SD = 0.660	M = 3.94 SD = 0.661	M = 3.90 SD = 0.659
	**E-SC**	M = 3.31 SD = 0.440	M = 3.43 SD = 0.407	M = 3.27 SD = 0.444
	**F-SC**	M = 4.36 SD = 0.626	M = 4.23 SD = 0.664	M = 4.41 SD = 0.605
	**P-SC**	M = 3.34 SD = 0.741	M = 3.64 SD = 0.681	M = 3.23 SD = 0.733

MD, Mediterranean diet; A-SC, academic self-concept; S-SC, social self-concept; E-SC, emotional self-concept; F-SC, family self-concept; P-SC, physical self-concept.

**Table 3:** Adherence to the MD according to self-reported physical activity level (*p* = 0.014).

MD	Physical Activity [% (*n*)]		Total [% (*n*)]
	Yes	No	
**Low adherence**	0.0% (*n* = 0)	1.0% (*n* = 3)	0.5% (*n* = 3)
**Medium adherence**	17.7% (*n* = 50)	25.7% (*n* = 81)	21.9% (*n* = 131)
**High adherence**	82.3% (*n* = 232)	73.3% (*n* = 231)	77.6% (*n* = 463)
**Total**	100.0% (*n* = 232)	100.0% (*n* = 315)	100.0% (*n* = 567)

**Table 4:** Dimensions of self-concept according to adherence to the MD.

Self-Concept	MD	M	SD	F	P
**A-SC**	**Low**	3.50	1.25	6.744	0.001
	**Medium**	3.45	0.62		
	**High**	3.67	0.58		
**S-SC**	**Low**	3.83	1.15	2.181	0.114
	**Medium**	3.80	0.62		
	**High**	3.94	0.66		
**E-SC**	**Low**	3.13	0.64	0.420	0.657
	**Medium**	3.29	0.40		
	**High**	3.31	0.45		
**F-SC**	**Low**	4.67	0.44	1.926	0.147
	**Medium**	4.28	0.69		
	**High**	4.39	0.60		
**P-SC**	**Low**	3.11	0.94	5.296	0.005
	**Medium**	3.16	0.73		
	**High**	3.39	0.73		

**Table 5:** Adherence to the MD according to religious belief, campus, and place of residence.

Variables		MD Adherence			*P*
		Low	Medium	High	
**Religious belief**	**Catholic**	0.6% (*n* = 2)	23.5% (*n* = 77)	75.9% (*n* = 249)	0.762
	**Muslim**	0.0% (*n* = 0)	22.7% (*n* = 22)	77.3% (*n* = 75)	
	**Atheist-Agnostic**	1.2% (*n* = 2)	20.1% (*n* = 33)	78.7% (*n* = 129)	
	**Other**	0.0% (*n* = 0)	25.0% (*n* = 2)	75.0% (*n* = 6)	
**Place Residence**	**Family Home**	0.8% (*n* = 3)	20.7% (*n* = 79)	78.5% (*n* = 299)	0.881
	**Shared Flat**	1.2% (*n* = 2)	25.5% (*n* = 45)	73.3% (*n* = 129)	
	**University Residence**	0.0% (*n* = 0)	20.0% (*n* = 8)	80.0% (*n* = 32)	
**Campus**	**Granada**	0.0% (*n* = 0)	9.8% (*n* = 10)	90.2% (*n* = 93)	0.030
	**Ceuta**	1.4% (*n* = 2)	23.9% (*n* = 33)	74.7% (*n* = 103)	
	**Melilla**	0.8% (*n* = 3)	26.7% (*n* = 95)	72.5% (*n* = 258)	
